# Social signals mediate oviposition site selection in *Drosophila suzukii*

**DOI:** 10.1038/s41598-021-83354-2

**Published:** 2021-02-15

**Authors:** Johanna E. Elsensohn, Marwa F. K. Aly, Coby Schal, Hannah J. Burrack

**Affiliations:** 1 Department of Entomology and Plant Pathology, NC State University, Raleigh, NC USA; 2 Department of Plant Protection, Faculty of Agriculture, Minia University, El-Minya, Egypt

**Keywords:** Agroecology, Behavioural ecology, Invasive species, Microbial ecology

## Abstract

The information that female insects perceive and use during oviposition site selection is complex and varies by species and ecological niche. Even in relatively unexploited niches, females interact directly and indirectly with conspecifics at oviposition sites. These interactions can take the form of host marking and re-assessment of prior oviposition sites during the decision-making process. Considerable research has focused on the niche breadth and host preference of the polyphagous invasive pest *Drosophila suzukii* Matsumura (Diptera: Drosophilidae), but little information exists on how conspecific signals modulate oviposition behavior. We investigated three layers of social information that female *D. suzukii* may use in oviposition site selection—(1) pre-existing egg density, (2) pre-existing larval occupation, and (3) host marking by adults. We found that the presence of larvae and host marking, but not egg density, influenced oviposition behavior and that the two factors interacted over time. Adult marking appeared to deter oviposition only in the presence of an unmarked substrate. These results are the first behavioral evidence for a host marking pheromone in a species of *Drosophila*. These findings may also help elucidate *D. suzukii* infestation and preference patterns within crop fields and natural areas.

## Introduction

During host finding for oviposition, female insects incorporate a number of information types into their decision making process^1–3^. Especially important for oviposition site selection (OSS) can be social information, particularly signals relayed by individuals of the same species. Whereas personal information is directly gathered by an individual assessing its environment, social information includes cues and signals provided by other organisms to reduce an individual’s uncertainty in that environment^1,4^. Females continuously gather and assess this information as they move through space and time, as a change in conditions might alter their behavior. Females need to be able to balance the cost of information gathering in time, energy, and risk of predation with its potential value in finding an optimal oviposition site. Biotic and abiotic factors can provide valuable information to an individual at both long and short ranges. Visual and olfactory cues can help guide insects at a distance, while a number of factors including temperature, humidity, host quality, natural enemies, and competition can inform OSS within a localized area^2,4,5^. While these conditions are dynamic and not wholly predictable, social signaling can be used by an individual to influence and bias the behavior of other organisms through this information sharing.

One means by which insects share information is through the use of marking pheromones. Marking pheromones are a broad class of chemical compounds insects use to communicate information to other individuals about the presence of the insect or its progeny relating to a valued resource^6^. The resource may be a nutritional source, oviposition site, or site of shelter. The two dominant examples of marking pheromones are aggregation pheromones and host marking pheromones (HMP). Among other uses, aggregation pheromones can lead to attraction of other conspecifics to communally exploit a resource, while HMPs are almost always used to mark host resources that already contain eggs and serve to deter future oviposition^7^.

*Drosophila suzukii* Matsumura is an invasive polyphagous pest that experienced a rapid range expansion beginning in 2008 from parts of east Asia to a near global distribution^8–11^. A wide and diverse host range, including cultivated and wild-growing fruits, give female *D. suzukii* a plethora of potential oviposition sites from which to choose^12–15^. Females deposit single eggs per oviposition site in different host fruit of the same species^16^; each oviposition site is evaluated through a walking and probing behavior pattern that may or may not lead to egg deposition, indicating females are making an assessment on a case-by-case basis. Mechanoreceptors are found on the ovipositor tip while chemoreceptors on the abdominal terminus may also be involved in the assessment^17,18^. In general, female *D. suzukii* prefer to oviposit in a substrate that is semi-firm, has a high carbohydrate-to-protein ratio, and low pH^18–21^. Although some fruit characteristics influence oviposition preference, the choice of oviposition sites does not always correlate to larval performance^22,23^. Fruit-associated microorganisms, such as yeast and bacteria, appear to influence adult attraction^24–26^ and are also linked to oviposition behavior, with some microbial species increasing egg laying^27^ whereas volatiles from others deter oviposition^28^.

Social facilitation and the use of marking pheromones exists in some drosophilid species and true fruit flies, and recent evidence suggests that an aggregation pheromone may be used by *D. suzukii* to influence conspecific oviposition^29^. In another *Drosophila* species, *D. melanogaster*, both males and females leave aggregation pheromones on oviposition substrates, presumably in response to the competition faced within their ecological niche^30,31^. *Drosophila melanogaster* are saprophytic and encounter competitors from other insects and fungi when exploiting their preferred ephemeral, rotting substrate^32,33^. Aggregation pheromones left behind by both adult sexes help reduce the negative effects from interspecific competition. Conversely, *D. suzukii* exploit the relatively under-utilized niche of ripening and ripe fruit^20^, and as such do not face the same interspecific pressures that make aggregation pheromones especially valuable.

In addition to marking pheromones, female *D. melanogaster* utilize other forms of social information, such as the presence of larvae or fecal deposits, to positively influence egg laying^25,34^. Behaviorally and ecologically, *D. suzukii* differ in significant ways from *D. melanogaster* and may actually share more life history traits in common with true fruit flies (Tephritidae) due to their shared resource niche. Social facilitation has been observed in certain frugivorous species, such as the Mediterranean fruit fly *Ceratitis capitata* wherein females oviposit more eggs in the presence of conspecifics than in solitary conditions^35^. In the laboratory, the oviposition rate of *D. suzukii* females is positively influenced by the presence of adults^36^. Still, *D. suzukii* differ from many tephritids in that they do not face the same extreme negative effects of larval competition at low densities^37^. To mitigate the high costs of larval competition, several fruit feeding tephritid fly species utilize HMPs to discourage conspecific and interspecific oviposition on previously infested fruit^38,39^. However, the use of HMPs in fruit-infesting flies is not universal^40,41^.

The goal of this study was to understand the behavioral significance of social information in *D. suzukii* in the context of OSS, as this phenomenon is not well understood and has yet to be fully explored in this species. We designed a series of behavioral experiments in a laboratory setting to test whether *D. suzukii* mark oviposition sites and if female *D. suzukii* discriminate between unoccupied (uninfested) fruit and fruit occupied by eggs or larvae. Our results contribute fundamental insights into OSS in insects and offer a promising avenue of research into a potential HMP for this global pest.

## Results

### Females prefer to oviposit in unmarked substrates

**Figure 1:**
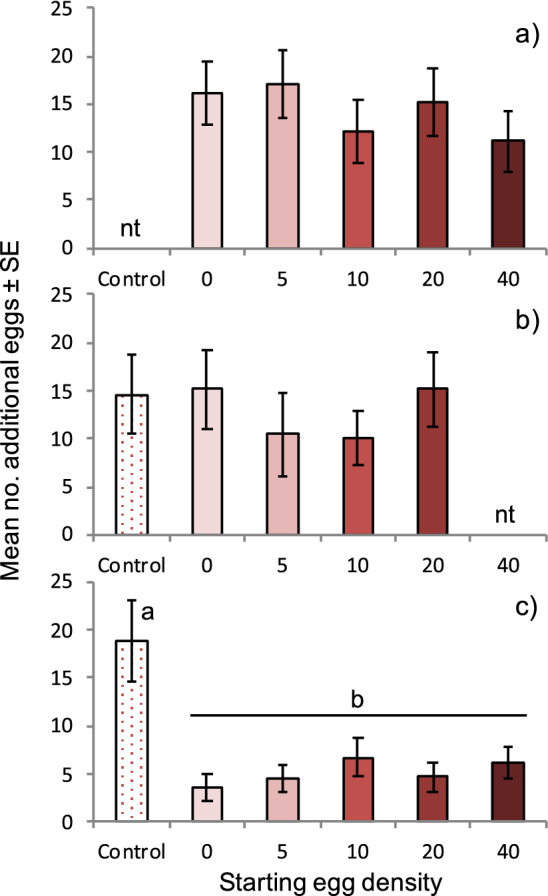
Female *D. suzukii* prefer to oviposit in unmarked substrates. Mean ± SE number of eggs oviposited per dish on a highly preferred raspberry juice-agar substrate in multi-choice assays. All dishes, except Control dishes, were exposed to adult *D. suzukii* for 2 h before the assay, and the starting egg density (per dish) was achieved by removing or adding eggs to dishes before the assay began. ‘Control’ is an unmarked, unoccupied dish. ‘nt’ = treatment was not tested. (**a**) All substrates were marked by adult *D. suzukii*, (**b**) All substrates were unmarked, but contained eggs if applicable (eggs were transferred from a marked to unmarked dish using sterile forceps), (**c**) Substrates except for the Control were marked by adult *D. suzukii*. Values sharing the same letter are not significantly different (alpha = 0.05).

Using a multi-choice behavioral assay, we exposed naïve females to several substrates that varied in egg density. To investigate whether visual or chemical cues were involved, we conducted multiple experiments using a raspberry juice agar substrate. The first experiment used substrates that were oviposited into directly by females (referred to as ‘marked’) and adjusted for egg number prior to exposure to naïve females. The second experiment transferred eggs from marked substrates into dishes that were not previously exposed to adult flies (referred to as ‘unmarked’). We found no relationship between the starting egg density and the number of new eggs deposited by *D. suzukii* females regardless of marking status (ANOVA, marked (Assay 1): F_4,40_ = 1.97, p = 0.118; unmarked (Assay 2): F_4,28_ = 0.533, p = 0.713; Fig. 1a,b). However, when females were given a choice between marked and unmarked substrates (Assay 3), more eggs were oviposited in the unmarked substrate (ANOVA, F_5,50_ = 8.608, p = 0.615 × 10^–6^; Fig. 1c), as measured by the 60% reduction in oviposition relative to the no-choice marked substrates in the first experiment.

**Figure 2:**
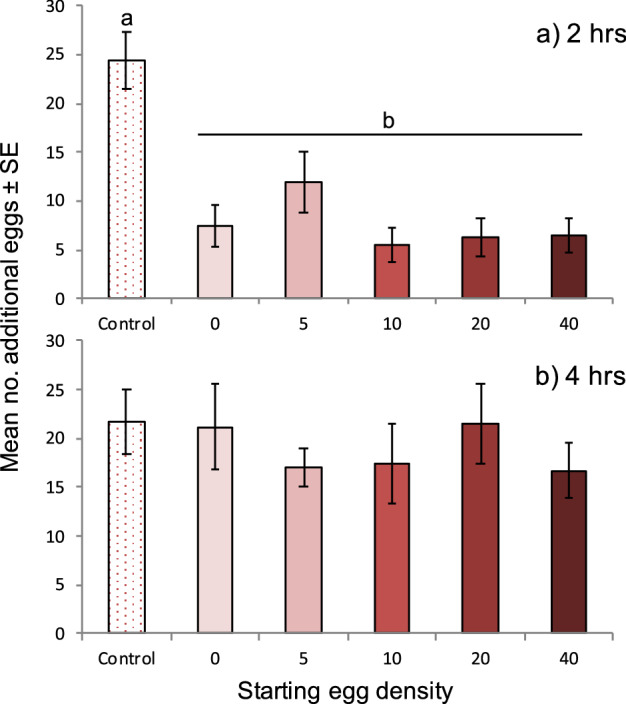
*D. suzukii* preference for unmarked substrates is modulated by host marking behavior during the assay. Mean ± SE number of eggs oviposited per dish on a less preferred raspberry puree-agar substrate in multi-choice assays. All dishes, except Control dishes, were exposed to adult *D. suzukii* for 2 h before the assay, and the starting egg density (per dish) was achieved by removing or adding eggs to dishes before the assay began. ‘Control’ is an unmarked, unoccupied dish. (**a**) 6-way comparison with dishes exposed for 2 h, (**b**) 6-way comparison with dishes exposed for 4 h. Values sharing the same letter are not significantly different (ANOVA, alpha = 0.05).

We tested this scenario again using a similar, yet lesser preferred substrate of raspberry puree agar made from blended whole fruit. The difference in preference between raspberry juice and puree was first examined by a 2-choice assay and showed that females laid approximately three times as many eggs in the juice agar than in the puree agar (raspberry juice: 10.33 ± 1.55 eggs ± SE, raspberry puree: 2.87 ± 0.96 eggs) (Wilcoxon, Z = 100, p = 0.003, df = 14). Using the puree substrate and the same set-up as in Assay 3, we observed a similar pattern as with the raspberry juice (ANOVA, Assay 4: F_5,45_ = 26.01, p = 3.03 × 10^–12^; Fig. 2a), suggesting that when provided a choice between marked and unmarked oviposition substrates, females direct their oviposition toward unmarked substrates and away from marked substrates, including marked substrates that contained no eggs. These results were confirmed in a follow-up 2-choice experiment, with the unmarked dish garnering more eggs than the marked dish (unmarked: 9.47 ± 1.49 eggs ± SE, marked: 4.87 ± 1.02 eggs); however, the difference was not significant (Wilcoxon, Z = 70, p = 0.589, df = 14).

Nevertheless, the observed preference for unmarked over marked substrates appears to be time-sensitive and limited only to choice scenarios. When the puree substrate assay was extended beyond 2 to 4 h, the difference between the unmarked and marked dishes disappeared (ANOVA, Assay 5: F_5,45_ = 0.509, p = 0.768; Fig. 2b), suggesting that once the preferred unmarked substrate becomes marked too, all other substrates become equally acceptable. This is illustrated through the number of eggs found in the control substrate; after a 2 h exposure approximately 24 eggs were counted, and that number did not increase during a 4 h exposure. A distinction between substrates was only noted when females were given a choice in either substrate type (juice or puree) or marking type (marked or unmarked).

### Marking by both sexes induces host rejection with selective use of larval cues

Due to the apparent influence of surface chemicals left behind by female *D. suzukii*, we next sought to test the influence of host marking and larval occupation at different development stages in a factorial design. We then tested these substrates against a control every 2 days after the initial set-up (marking and egg deposition) which coincided with the three larval instar stages of *D. suzukii*; day 0 tested the egg stage, day 2 first instars, day 4 second instars, and day 6 tested third instars. In each of the two-choice assays described hereafter, the treated substrate was always paired with an untreated (unmarked, unoccupied) control substrate of the same age.

**Figure 3:**
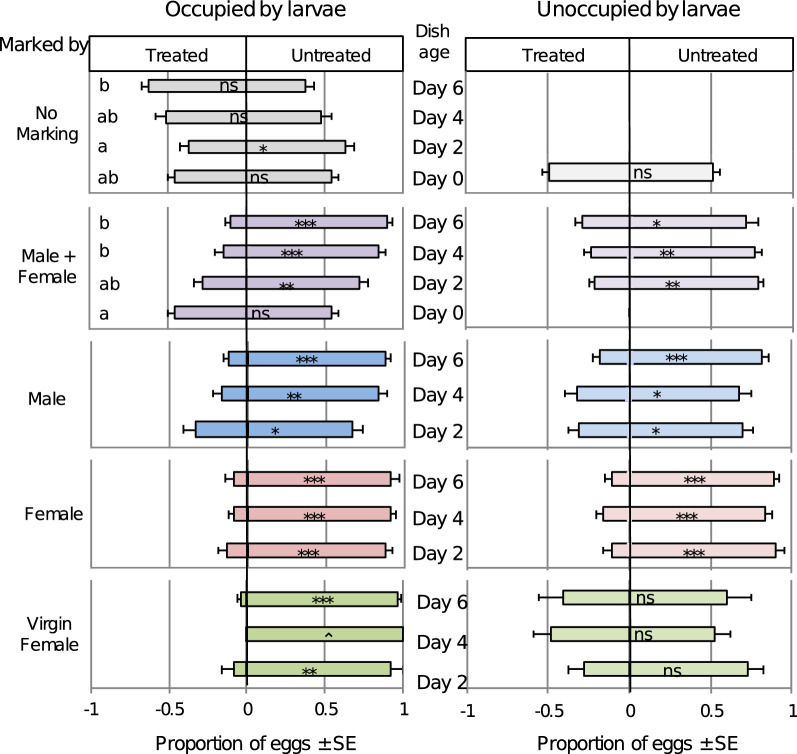
Larval density and host marking type affect *D. suzukii* oviposition preference. Data are presented as proportion of eggs laid in each dish ± SE. ‘Treated’ = marked and/or occupied substrate, ’Untreated’ = unmarked, unoccupied substrate. All dishes, except untreated dishes, were exposed to 10 *D. suzukii* adults for 4 h, followed by adding or removing eggs for the ‘occupied’ treatment. Dishes were either assayed again immediately, or held for a number of days prior to assay. Bars that share the same letter within each panel are not significantly different (Kruskal Wallis, Dunn Test, p > 0.05). Asterisks indicate significance values as follows: * p < 0.05; ** p < 0.01; *** p < 0.001 (Paired t-test). ⌃ No eggs were counted on the marked substrate.

Larval occupation and the presence of substrate marking from adults of both adult sexes together had a significant negative effect on oviposition (Kruskal Wallis, Occupied/Male + Female: χ^2^ = 23.96, p = 2.55 × 10^–5^, n = 78; Fig. 3), with females laying a greater portion of eggs on the control untreated dish in the presence of a marked substrate on days 2 through 6. When host marking was absent, but larvae were present in the treated substrate, there was no clear preference except on the second day, or first instar stage, when the treated dish received significantly more eggs than the control substrate (Wilcoxon, Occupied/No Marking: Z = 132, p = 0.045, n = 20; Fig. 3).

Oviposition preference was affected by the larval development stage only when males were included in the marking treatment. We observed a general trend that the preference became stronger over time, coinciding with later instars (Kruskal Wallis, Occupied/Male + Female: χ^2^ = 23.96, p = 2.55 × 10^–5^, n = 78; Occupied/Male: χ^2^ = 6.80, p = 0.035, n = 60; Fig. 3). In other words, if oviposition was deterred by the presence of first instars (day 2) in the treated dish, deterrence was also observed and was numerically higher on days 4 and 6 with later instar larvae. Similar to the results from the egg density experiments, females showed no oviposition preference on day 0 when the egg stage was tested (Wilcoxon: Occupied/Male + Female: Z = 103.5, p = 0.446, n = 19; Occupied/No Marking: Z = 105, p = 0.408, n = 20; Fig. 3).

Host marking by mated females produced the highest deterrence of egg laying and did not significantly vary over time or due to larval occupation (Kruskal Wallis, development stage: χ^2^ = 0.658, p = 0.720, df = 2; larval occupation: χ^2^ = 1.65, p = 0.199, df = 1; n = 119; Fig. 3). Although larvae increased deterrence in male and combined sex trials, there was no significant difference between occupied and unoccupied treatments within a marking type. The combination of marking by virgin females and presence of larvae elicited strong deterrence of oviposition (Kruskal Wallis, Occupied/Virgin Female: χ^2^ = 25.216, p = 5.125 × 10^–7^; Fig. 3). However, females exposed to a substrate marked by virgin females on unoccupied dishes (no larvae) showed little to no preference (Kruskal Wallis, Unoccupied/Virgin Female: χ^2^ = 2.635, p = 0.268; Fig. 3), suggesting that marking by mated females may be qualitatively or quantitatively more deterrent than marking by virgin females, which lay few eggs.

## Discussion

Female insects have many sources of information available when choosing where to oviposit their eggs in a dynamic landscape. We tested three potentially important sources of social information they likely encounter. By manipulating egg density, larval age and potential host marking pheromones on the oviposition substrate, we were able to show that *D. suzukii* adults leave behind a signal that deters oviposition by naïve females, but only when an unmarked substrate was also available. Egg density did not affect the degree of deterrence. Presence of larvae also deterred egg laying in a larval age-dependent manner.

Host acceptance in generalist species is in part constrained by deterrent compounds found in plants^42^. The presence of conspecific deterrent signals left behind by individuals on plant tissue may act to further narrow host choice by guiding OSS. Recognition of signals that indicate prior visitation to a host guides more informed oviposition choices in several insect orders^40,43^. In our research, females did not seem to recognize egg-related cues but avoided larvae-infested, marked substrates, with greater deterrence observed with later larval stages. These behavioral findings, combined with competition-induced larval mortality^37^, suggest that female *D. suzukii* are using social information to select unmarked, unoccupied substrates for oviposition, when available. Our results also indicate that host marking may be a more reliable source of social information for OSS than larvae. When both information types were present, they worked in concert to cause significant oviposition deterrence. However, marking alone was sufficient to deter females from ovipositing on the marked substrate even in the absence of any larvae. Conversely, females were not deterred from unmarked substrates occupied by larvae, and even slightly attracted in 2-choice assays by the presence of late instar larvae. As larvae develop within a resource, they are processing and altering its nutritional quality, making the substrate more or less suitable for subsequent visitors. While this may not happen in natural settings, our experiments suggest that females can use larval cues as a signal of host quality absent any other social information.

Like other frugivorous species, such as tephritids, *D. suzukii* face greater conspecific than interspecific competition. Within large monocultures that are common on farms, *D. suzukii* females have ample oviposition site choices within a relatively small area. Because their preferred oviposition substrate of ripe fruit is ephemeral, however, females need to quickly identify a host that can support larval development through pupation before later colonizers (e.g., *D. melanogaster*) start to outcompete slower developing *D. suzukii* larvae^44^. Our results support the hypothesis that *D. suzukii* females and males use host marking pheromones, which enforce skip oviposition and maximize larval fitness^16^.

The use of host marking information by *D. suzukii* appears to be highly context-dependent. In the egg density experiment, when gravid females were given a choice of substrates that were all one type (e.g., marked or unmarked, juice or puree), females oviposited a similar number of eggs in the different treatments, independent of the density of previously oviposited eggs they encountered. However, whenever females had a choice between a marked and unmarked substrates, or between juice and puree, their oviposition was strongly biased based on specific preferences. In 2-choice tests, females oviposited twice as many eggs in unmarked dishes than in marked dishes, and three times as many eggs in raspberry juice-agar substrates than in raspberry puree-agar. Preference for unmarked dishes was even stronger when marking was allowed to age, with females ovipositing over ninefold more eggs in the unmarked than marked substrates.

As a time-constrained and highly polyphagous species, *D. suzukii* females have short time windows to process available information and make an oviposition decision among a myriad choices^45^. HMPs are typically drawn on the fruit surface and used by individuals to quickly identify oviposition sites previously exploited by themselves or conspecifics^40,41^. In the 2 h multi-choice egg density assays, females changed their oviposition behavior depending on the marking status of all offered substrates. Based on these lab observations, if all available wild or cultivated hosts within an area are marked, we would expect high numbers of eggs per fruit as females would exclude host marking from their oviposition decision. Indeed, during times of high *D. suzukii* population levels females oviposited more than 12 ± 0.36 eggs per gram of blackberry in wild fruits compared to < 5 ± 0.27 eggs/g in cultivated blackberries^36^. The hypothesis that females exclude host marking from their oviposition decision when unmarked fruit are unavailable is further supported when comparing the 2 and 4 h assays. In 2 h assays, females oviposited significantly more in the control unmarked substrate than in marked substrates containing various densities of eggs. However, in 4 h assays, females distributed their eggs across all substrates, independent of marking and prior egg density. It appears that at some point between 2 and 4 h, the unmarked control substrate became sufficiently marked to obscure any differences among the various treatments. It is important to consider, however, that in the field females have the option to fly away from marked fruit, or a patch that contains marked fruits, and seek unmarked fruits.

Our findings appear to conflict with the results of Tait et al.^29^. In a multi-choice test, marked blueberries at the highest egg density (> 10 eggs/berry) treatment received a greater number of eggs from naïve females than lower egg density, unmarked berries with simulated ovipositor punctures, or control blueberries after a 2 h exposure. During the experimental set-up, the berries for the marked plus egg treatments were exposed to female *D. suzukii* for 2 h. Instead of attraction, our results showed the opposite effect, whereby the marked substrate was less preferred for oviposition over an unmarked substrate in 2- and multi-choice scenarios. This seeming discrepancy may be explained by our observations of context-dependence. We used a highly preferred host fruit, raspberry, in an agar-based medium, eliminating penetration force as a potential barrier to oviposition. Raspberries are more preferred for oviposition and are a better nutritional resource for larvae than blueberry fruit^46^. If females incorporate host type and nutritional information into OSS decision making, we might expect a change in their behavior, which could explain the conflicting preference results^47^. Expanding *D. suzukii* host marking research into other host species will help better understand the contexts where females utilize social information in OSS. Interestingly, Tait et al.^29^ found no differences in egg laying between the treatments during subsequent time assessments (4, 24, and 48 h), coinciding with our results that suggest temporality may be an important consideration.

Further research is needed to isolate and identify what types of compounds female and male *D suzukii* leave behind on marked substrates. The deterrent marking compounds we observed were stronger from females but present in both sexes and may be a mix of feces, regurgitant, or other excretions. These compounds could be related to mating or mated status, as marking by virgin females in unoccupied dishes did not deter oviposition. Cuticular hydrocarbons (CHCs) are important in mate recognition and host marking in *D. melanogaster* as well as in tephritids^25,31,48^. CHCs in both sexes of adult *D. suzukii* have the same composition but compounds differ in relative abundance between males and females, which may underlie the differences in deterrence we observed between male and female marking^49^. Because we chose to focus solely on the behavioral aspect of oviposition deterrence for these experiments, we cannot make any further deductions at this time.

The putative HMP suggested here may be of microbial origin. Many insect species use fecal-derived volatiles as pheromones or allelochemicals [e.g.,^50–52^]. Research on *D. suzukii*-derived microbes from adults and infested fruits confirms their role in adult attraction^53^. We observed microbial growth on the marked dishes beginning on the second day post marking and infestation. Microbial volatiles strongly influence oviposition behavior in *D. melanogaster* and might mediate the observed oviposition deterrence in this study in *D. suzukii*. Both environment and diet directly influence *Drosophila* gut microbial community composition and abundance^54,55^. Since gut microbiota differ between and among lab and wild *D. suzukii* populations^56,57^, further research will need to evaluate the influence of social information using wild flies and in field settings where host marking would only be one of a variety of information sources used in OSS.

Our results indicate that conspecific host marking significantly reduces, but does not eliminate oviposition by naïve females. Our research provides strong evidence for the use of an HMP in *D. suzukii*. While HMPs have been proposed as a pest management tool for other fruit-infesting flies^58^, existing methods may not work universally to reduce *D. suzukii* oviposition given the importance of context on OSS shown in our data. However, the behavioral insights into decision making under choice and no choice scenarios suggest there may be potential in exploiting this information in push–pull or trap crop strategies, as suggested by others^59,60^. A clear next step in this line of research will be to identify and isolate the activating, deterrent compounds.

## Methods

### Flies

A laboratory colony of *D. suzukii* was established from wild-infested fruit in 2011 and maintained on a cornmeal and yeast diet^37^. The colony was genetically augmented periodically with wild-collected *D. suzukii*. Flies were maintained at 20 ± 3 °C and 65 ± 10% relative humidity on a 12L:12D photocycle. Flies used in all experiments were 5–9 days old and females were presumed mated at this age^61^.

### Oviposition substrates

We used two raspberry-based agar substrates for oviposition assays. A “raspberry puree” substrate was made by blending whole, ripe raspberries (Driscolls organic, USA origin) with equal parts water, and 1% each of agar, and the anti-fungal agents: Tegosept (methyl paraben [CAS: 99-76-3]), and propionic acid (CAS:79-09-4). Approximately 20 ml of this mixture was added to 35 × 10 mm Petri dishes (Falcon, Corning Incorporated, Durham NC) and set. A “raspberry juice” substrate was made by hand straining fresh, ripe raspberries through a layer of fine mesh to separate the juice from the pulp and seeds; the juice was then used in the above recipe in place of the puree. Water was boiled with agar for approximately 1 min, then removed from heat and poured into a container holding the raspberry puree/juice. This cooled the solution enough to add the anti-fungal agents. The solution was mixed after each ingredient addition.

While no exogenous microorganisms were added during these experiments, they may have been present on raspberry fruit at the time of purchase. Fruit were not cleaned prior to use, and were not boiled to conserve as much of the ambient microbial community as possible. As some of the experiments lasted up to six days, Tegosept and propionic acid were used as microbial inhibitors to prevent overgrowth of any pre-existing microorganisms, whether pathogenic or beneficial. The addition of these chemicals did not appear to prevent the establishment and proliferation of microorganisms left on the surface of the fruit agar by adults. These microbial inhibitors have been used in *D. melanogaster* studies where microbes were tested for pathogenicity^62^ and their role in marking behavior^31^.

Preliminary assays to directly compare the difference between substrates indicated a 3:1 oviposition preference for the juice agar over the puree in a 2-choice assay, where 5 pairs of *D. suzukii* adults were exposed to single juice and puree substrate in a 473-ml assay container for a period of 2 h. Ultimately, both substrates were used in the following experiments as the juice was more preferred for egg laying while the puree is likely more similar texturally to natural whole fruit substrate in which *D. suzukii* oviposits.

### Experiment 1. Host marking and egg density

#### Exp. 1. Set-up and pre-infestation

To obtain various egg densities (0, 5, 10, 20, 40 eggs), 25–30 raspberry juice agar dishes were placed in a 0.3 × 0.3 × 0.3 m cage with approximately 200 adult females and males. Dishes were removed after 2 h and the eggs per dish were counted. Dishes with the largest number of eggs laid were used for the highest density treatment. We attempted to manipulate the density as little as possible, but when necessary, eggs were removed with fine-point forceps or they were gently transferred with sterile forceps and laid on or under the surface to mimic natural oviposition. These dishes were used in the following bioassays the same day, as the majority of *D. suzukii* eggs hatch within 24 h of oviposition^63^.

#### Exp. 1. Behavioral assays

Twenty females were placed in a 0.3 × 0.3 × 0.3 m cage and exposed to five raspberry juice substrates simultaneously of the following densities: 0, 5, 10, 20, and 40 eggs per dish (Assay 1). Dishes were placed in a 2 × 3 block in the center of the arena, with the position of each treatment randomized within the cage. After a 2 h exposure, all dishes were removed, and the eggs on each dish were counted. The starting egg density number was subtracted from the final egg count to obtain the number of additional eggs laid during the assay.

Substrates in Assay 1 were marked by adult flies during the original infestation period. To isolate the effect of egg density alone, we gently transferred eggs from exposed to unexposed juice agar substrates at the same densities as in the first experiment: 0 (undamaged), 0 (damaged), 5, 10, or 20 eggs per dish, omitting the 40 egg/dish treatment (Assay 2). Eggs were transferred by placing the tip of sterilized fine needle forceps under the egg and gently lifting the forceps up, which detached the egg from the oviposition site and transferred the egg to the forceps. This method did not transfer any agar from the marked substrate (that could be observed at 40× magnification), and it also limited, but did not eliminate potential contamination from compounds on the surface of the marked substrate. We did not wash the eggs because we wanted to assess any cues directly associated with an egg, including chemical cues. To mimic egg removal from the other assays, the surface of the “damaged” zero-egg-treated dishes was mechanically damaged with sterile forceps. We added twenty females to a 0.3 × 0.3 × 0.3 m cage with the randomized blocked substrates for a 2 h exposure.

To test the effect of host marking—hereafter referred to as “marked”—by adult females and males, we conducted a separate experiment that included a treatment consisting of an additional dish that had not been exposed to adults (unmarked and contained no eggs). This treatment was tested in a six-way comparison with the five previously described treatments using the same methodology (Assay 3). We also repeated this experiment using the less preferred whole fruit puree substrate (Assay 4). To assess the effect of exposure time, we ran additional replicates of the puree assay for 4 h, or double the time of the other assays (Assay 5).

In response to a seemingly large influence of host marking in our first series of experiments, we conducted an additional experiment to directly compare oviposition preference between marked and unmarked substrates. We created the marked dishes by exposure to approximately 200 flies as done during set-up. Any eggs laid in these dishes were counted and removed. A single marked and unmarked raspberry juice agar dish were added to a 473-ml plastic assay container along with 5 male and 5 female *D. suzukii* for 2 h.

### Experiment 2. Host marking and larval development

#### Exp. 2. Set-up and pre-infestation

To test the effect of any materials associated with walking, defecation, and active marking by both sexes, we exposed a single puree agar dish to 5 male and 5 female *D. suzukii* in a 473-ml arena. Dishes were removed after 4 h and the eggs counted. The oviposition propensity of females can vary widely between small cohorts of flies during time-limited assays, so to standardize the larval density we redistributed eggs so that every marked dish had a density of 10 eggs to assess effects of larval development and resource use on female attraction and oviposition. Prior to testing, marked and unmarked (control) dishes were kept in individual plastic vented containers in a growth chamber (25 ± 2 °C, 70 ± 10% RH, 12L:12D photocycle).

To remove the effect of adult marking, we stretched Parafilm (Bemis Company, Inc., Neenah WI) over the substrate surface, while still allowing for successful oviposition. A 2.5 × 2.5 cm section of Parafilm was stretched to ~ 6.5 × 6.5 cm and placed over each dish. Females could penetrate the film to lay eggs, but the physical barrier prevented any materials other than those immediately associated with oviposition from accumulating on the substrate. Notably, however, the oviposition holes could serve as feeding sites for adult males and females at this time. In the assays where no marking was present, no microbial growth was observed.

A single Parafilm-covered dish was exposed to 5 male and 5 female *D. suzukii;* females were allowed to oviposit for 24 h instead of only 4 h because of increased latency to oviposition due to the lack of tarsal contact with the substrate caused by the film barrier. The dish was then removed, and the number of eggs per dish recorded. Again, eggs were redistributed when necessary so that each dish had a final count of 10 eggs per dish. Control dishes were treated in the same manner (Parafilm covering, placed in assay container for 24 h) except no flies were added. Dishes were then stored in individual plastic vented containers in a growth chamber until needed.

To test the effects of adult marking on subsequent oviposition preference, in the absence of eggs or larvae, five pairs of adults were allowed to explore and oviposit on uncovered petri dishes for 4 h. Eggs were counted and removed. To assess sex-specific marking, we conducted follow-up experiments using 10 each of males, mated females, and virgin females in place of the five adult pairs during the host marking phase. Egg-containing dishes for the male and virgin female occupied treatments were generated using Parafilm-covered dishes to minimize any female surface marking, followed by a 4 h exposure to males or virgin females for marking. Virgin females did not oviposit unfertilized eggs during the marking period. For the female unoccupied treatment, eggs laid during the marking phase were counted and removed. All other experimental aspects remained the same as described previously.

#### Exp. 2. Behavioral assays

*Effects of marking, egg and larval development on oviposition preference*

We compared egg laying in egg and larval dishes every 2 days to test the effect of each developmental stage on egg laying preference with each dish only used for a single assay. In a separate, preliminary experiment we determined the time needed post-oviposition for the majority of larvae to be in the desired life stage on the day of testing, confirming results observed in Emiljanowicz et al.^63^. Oviposition preferences were assayed in response to (a) eggs on the day they were laid (day 0), (b) first instars on day 2, (c) second instars on day 4, and (d) third instars on day 6. On the day 2, egg chorions and any unhatched eggs were removed from all infested dishes so that only newly oviposited eggs would be counted during subsequent assays on days 2–6.

For each time point (day 0, 2, 4, or 6), marking treatment (presence or absence of marking) and larval occupation (presence or absence of eggs or larvae), a 2-choice assay compared a ‘treated’ dish versus an ‘untreated’ control dish in a 473-ml arena. Five adult male and five adult female *D. suzukii* were added to the container along with the two substrates and assayed for 4 h, alternating the locations of treated and control dishes between replicates to control for potential side bias. We chose a 4 h assay period to standardize our lab methodologies with prior preliminary experiments. At the end of the assay, both dishes were removed, eggs were counted and occupied dishes were carefully dissected to assess the number and stage of surviving larvae.

### Data analysis

#### Exp. 1. Host marking and egg density

We used an analysis of variance (ANOVA) test to look for significant differences between the number of eggs laid and the original density count, with replicate as a random effect. If a significant difference was detected, Tukey’s HSD was used for mean separation.

#### Exp. 2. Host marking and larval development

To normalize variation between replicates, we calculated the proportion of eggs laid in the treated or control dishes (*number of eggs in treated or control / total egg count*) and excluded any non-responders from analysis (6 of 465 total replicates). We then used the Wilcoxon test to compare the proportion of eggs laid in the control dish against the null hypothesis of a 50:50 distribution. The Kruskal–Wallis test was used to individually assess the effect of larval presence and age within each host marking treatment on the proportion of eggs in the control dish with a Dunn test used to separate means when appropriate. All analyses were conducted in R v. 3.6.1^64^.

## Data Availability

The datasets generated during and/or analyzed during the current study are available from the corresponding author upon reasonable request.
